# Aging Impairs the Proliferative Capacity of Cardiospheres, Cardiac Progenitor Cells and Cardiac Fibroblasts: Implications for Cell Therapy

**DOI:** 10.3390/jcm2030103

**Published:** 2013-09-23

**Authors:** Jianqin Ye, Douglas S. Hom, Joy Hwang, Yerem Yeghiazarians, Randall J. Lee, Andrew J. Boyle

**Affiliations:** 1 Department of Medicine, Division of Cardiology, University of California San Francisco, San Francisco, CA 94143, USA; E-Mails: jianqiny@medicine.ucsf.edu (J.Y.); douglas.hom@gmail.com (D.S.H.); joy.hwang.ucsf@gmail.com (J.H.); yeghiaza@medicine.ucsf.edu (Y.Y.); lee@medicine.ucsf.edu (R.J.L.); 2 Edyth and Eli Broad Center for Regeneration Medicine and Stem Cell Research, University of California San Francisco, San Francisco, CA 94143, USA

**Keywords:** cardiac progenitor cell, aging, cardiac fibroblast, proliferation, paracrine signaling

## Abstract

**Introduction:** Cardiospheres (CS) are self-assembling clusters of cells that can be grown from cardiac tissue. They contain a heterogeneous cell population that includes cardiac progenitor cells (CPCs) and cardiac fibroblasts. CS and CPCs have been shown to improve cardiac function after myocardial infarction (MI) in experimental models and are now being studied in clinical trials. The effects of aging on the proliferative capacity of CS and CPCs, and the paracrine signaling between cell types, remain incompletely understood. **Methods and Results:** We compared the growth of CS from young and aging murine hearts at baseline and following MI. The number of CS from young and aging hearts was similar at baseline. However, after MI, young hearts had a dramatic increase in the number of CS that grew, but this proliferative response to MI was virtually abolished in the aging heart. Further, the proportion of cells within the CS that were CPCs (defined as Sca-1(stem cell antigen-1)^+^/CD45^−^) was significantly lower in aging hearts than young hearts. Thus the number of available CPCs after culture from aging hearts was substantially lower than from young hearts. Cardiac fibroblasts from aging hearts proliferated more slowly in culture than those from young hearts. We then investigated the interaction between aging cardiac fibroblasts and CPCs. We found no significant paracrine effects on proliferation between these cell types, suggesting the impaired proliferation is a cell-autonomous problem. **Conclusions:** Aging hearts generate fewer CPCs, and aging CPCs have significantly reduced proliferative potential following MI. Aging cardiac fibroblasts also have reduced proliferative capacity, but these appear to be cell-autonomous problems, not caused by paracrine signaling between cell types.

## 1. Introduction

Aging is associated with a higher incidence of myocardial infarction (MI) [1], and higher prevalence of heart failure in those that survive MI [2]. The cellular and molecular mechanisms that underlie the exaggerated left ventricular (LV) remodeling in aging patients remain incompletely described [3]. One potential mechanism that may contribute to post-infarction heart failure in the elderly is stem cell failure [4].

We have previously grown cardiospheres (CS), self-assembling clusters of cells, from murine hearts [5,6]. CS contain multiple cell types including cardiac progenitor cells (CPCs) and cardiac fibroblasts [7]. It is thought the heterogeneous nature of the cells contained within CS recapitulates the *in vivo* stem cell niche, conferring a benefit upon CS over other cell types as a source for cell therapy to achieve myocardial regeneration [7]. Following MI, we have shown that the proliferation rate of CS dramatically increases, with the proportion of CPCs within the CS remaining constant, and that CPC derived from CS can reduce scar size and improve function in the infarcted heart [5]. However, what happens to the growth rate of CS in aging hearts, particularly after MI remains unknown. Recently, cells derived from CS have been used in early clinical trials [8]. Because MI in humans is a disease-state that is strongly associated with aging, it is paramount to understand the effects of aging and MI on CS and CPCs.

Cardiac fibroblasts constitute a large proportion of the cells within the adult heart [9], are part of cultured cardiospheres [10], and are known to become dysfunctional with aging [11]. They therefore represent a potential cell of interest that may interact with and influence the behavior of CPCs. In this series of *in vitro* experiments, we demonstrate an age-related impairment of CS growth, a reduction in the number of CPCs, and we investigate whether this is due to interactions between aging cardiac fibroblasts and CPCs.

## 2. Materials and Methods

All procedures were approved by the UCSF Institutional Animal Care and Use Committee.

### 2.1. Cardiosphere Culture

Sca-1^+^/CD45^−^ cardiac progenitor cells were isolated from male C57BL/6 mice as we have described previously [5,6], based on the original methods used by Messina *et al*. [12]. For this series of experiments, young mice were 3 months of age and aging mice were 18 months old. The whole heart was removed from the mice and cut into 1–2 mm^3^ pieces. After being washed with Ca^++^ Mg^++^ free phosphate-buffered saline (PBS) and digested three times, 5 min each at 37 °C with 0.25% trypsin (Invitrogen, Carlsbad, CA, USA) and 0.1% collagenase D (Roche Diagnostics, Indianapolis, IN, USA), the tissue pieces were cultured as “explants” on fibronectin (Sigma, St. Louis, MO, USA) coated 6-well plates, 2 wells for each heart in Iscove’s modified Dulbecco’s Medium (IMDM) with 10% fetal bovine serum (FBS) and 0.1 mM β-mercaptoethanol at 37 °C with 5% CO_2_. A layer of fibroblast-like cells grew from explants, over which small, round phase-bright cells (CS-forming cells) appeared 2 to 4 weeks after initiating the culture. Once the fibroblast-like cells grew to 90% confluence determined visually, the cells surrounding the explants were harvested by two washes with PBS, one wash with 0.53 mmol/L EDTA and one wash with 0.05% trypsin (Invitrogen) at room temperature. The harvested cells were filtered by 70 mm cell strainer (BD Biosciences, San Jose, CA, USA), and then cultured at a density of 1 × 10^5^ cells/mL in each well of 24-well plates coated with Poly-d-Lysine (BD Biosciences) in cardiosphere growth medium (CGM), which included 35% IMDM, 65% DMEM-F12, 3.5% FBS, 0.1 mM β-mercaptoethanol, 2% B27 (Invitrogen), 10 ng/mL epidermal growth factor (R & D systems), 20 ng/mL basic fibroblast growth factor (R & D systems), 40 nmol/L thrombin (R & D systems) and 4 nmol/L cardiotrophin (R & D systems). The number of CSs in each well was counted by visual inspection.

### 2.2. Flow Cytometry

CSs were dissociated into single cell suspension by Blendzyme 4 (5.6 u/mL) (Roche). The following phycoerythrin (PE) or allophycocyanin (APC) conjugated rat anti-mouse antibodies and conjugated isotype-matched control antibodies were used: Sca-1-PE, c-kit-PE, CD133-PE, CD34-PE, CD45-APC, Flk-1-APC and CD31-APC (eBioscience, San Diego, CA, USA). The cells were incubated with antibodies for 25 min on ice, washed with PBS containing 0.2% BSA, and analyzed by FACSCabilur with CellQuest software (BD Biosciences).

### 2.3. Myocardial Infarction

MI was induced surgically by a permanent ligation of the left anterior descending (LAD) coronary artery as we have previously described [5,13]. Briefly, with the animal anesthetized and ventilated, permanent ligation of the LAD is made by a 7–0 suture in the anterior myocardium at 50% of the length of the heart from the anterior-inferior edge of the left atrium to the apex. The chest is then closed and the animal allowed to recover. One week after MI, hearts were harvested for CS culture.

### 2.4. Cardiac Fibroblast Isolation and Primary Culture

Cardiac fibroblasts were isolated from young and aging mice. Whole hearts were removed, placed in cold serum-free Dulbecco’s modified Eagle medium (DMEM; UCSF Cell Culture Facility, San Francisco, CA, USA), and perfused with 2 μg/mL collagenase II in Hank’s buffered salt solution. The left ventricle was dissected away, cut into small pieces, and incubated with the collagenase solution at 37 °C for 10 min. The supernatant was removed and the tissue was incubated with fresh collagenase again to improve yield. The cell suspension was passed through a 100 μm cell strainer to remove undigested tissue. The filtrate was centrifuged at low speed for 2 min to remove cardiomyocytes and then at a higher speed for 10 min to pellet smaller cells. The cells were plated with to allow fibroblasts to attach. After 1 h, medium was changed to remove non-adherent cells. The cells were cultured in DMEM supplemented with 10% fetal bovine serum. Medium was changed every two days.

### 2.5. Co-Culture of CPCs and Cardiac Fibroblasts

To assess the rate of proliferation of cardiac fibroblasts, 5000 fibroblasts were plated in each well of 24-well plates. 10,000 Sca-1^+^/CD45^−^ CPCs, or control medium, were seeded in inserts within the wells of the plate. Cardiosphere growth medium was used.

To assess the rate of proliferation of CPCs and the interaction with cardiac fibroblasts, CPCs were plated in the wells and cardiac fibroblasts in the inserts. Cardiac fibroblasts (15,000 cells/insert) were first plated in excess of CPCs (10,000 cells/well), and in the second experiment the CPCs (50,000 cells/well) were in higher numbers than cardiac fibroblasts (5000 cells/insert).

For all co-culture experiments, cell proliferation was measured by incubating with MTS (Promega, Madison, WI, USA) for 3.5 h. Absorbance at 490 nm was recorded using a plate reader. Proliferation is measured in relative light units (RLU) and then normalized to the appropriate control for each experiment (*i.e*., the first column in the graph). 

## 3. Results

### 3.1. Reduction in Cardiosphere Growth and Cardiac Progenitor Cells with Aging

**Figure 1:**
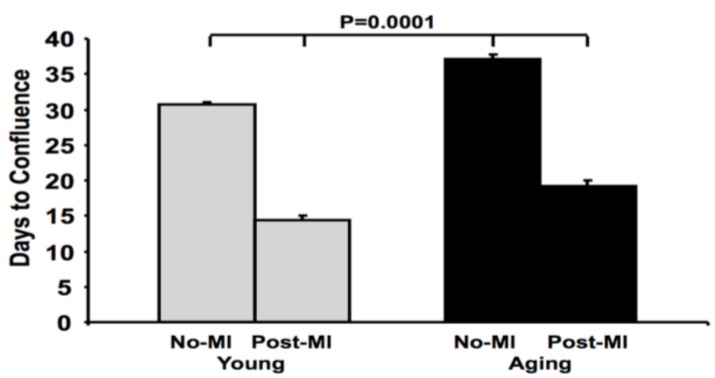
Explants from aging hearts grow more slowly. Explants from hearts of aging mice grow to confluence *in vitro* more slowly both at baseline and after MI. The aging hearts retain some ability to increase the explant outgrowth rate.

To investigate the effects of aging on the resident cardiac progenitor pool following MI, whole hearts were removed from young and aging mice, and cut into small pieces as “cardiac explants”. A monolayer of fibroblast-like cells migrated out from the cardiac explants over several weeks in culture. From this monolayer, small, round, phase-bright cells (CS-forming cells) were seen to emerge. We observed that aging cardiac explants took longer time to form a confluent monolayer than young explants in culture (*p* < 0.001) (Figure 1). We also found that explants from infarcted hearts (1-week post-MI) grew faster than those from non-infarcted hearts in both young and aging mice (*p* < 0.001), however post-MI explants from aging hearts grew slower than those from young post-MI hearts (*p* < 0.001, Figure 1).

**Figure 2:**
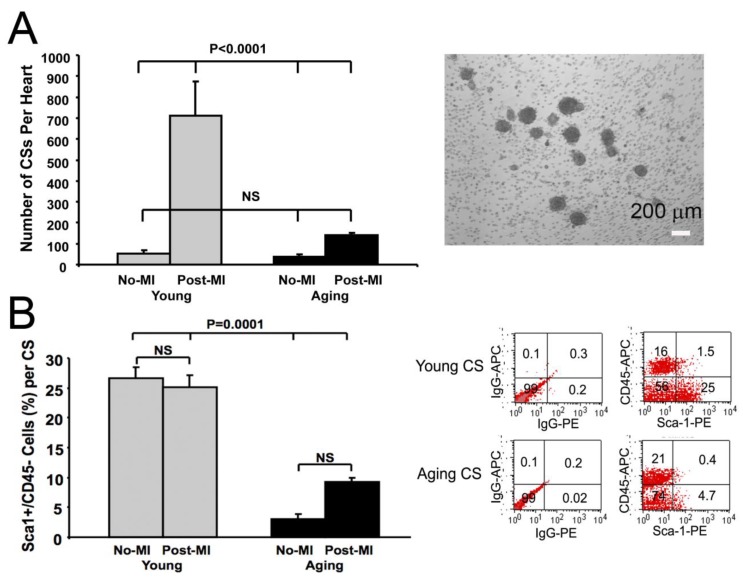
Age limits the proliferative capacity of cardiac progenitors following MI. (**A**) The number of CSs from young and aging murine hearts at baseline was not significantly different. The number of CSs from injured young hearts (*n* = 10) was much more than those from non-injured young hearts (*n* = 5). Aging blunts the proliferative response seen after MI. The number of CS generated from non-injured (*n* = 16) or injured (*n* = 13) aging hearts is similar. Cardiospheres are seen as self-assembling clusters of cells; (**B**) Bar graph showing the percentage of Sca-1^+^/CD45^−^ cardiac progenitor cells within CSs from young or aging hearts at baseline or 1-week post-MI by FACS (*n* = 5–8). Data in figure shown as mean ± SEM.

We observed that the total number of cardiospheres (CSs) derived from each heart in young and aging mice at baseline were not significantly different (Figure 2). However, the number of CSs derived from injured hearts was much higher than from non-injured hearts in young mice (*p* < 0.004), but not in aging mice (*p* = ns). These results suggest that the number of cardiac progenitors resident within the heart is not significantly decreased at baseline with age; however the ability of CPCs to proliferate in response to acute injury is impaired in the aging heart. 

We analyzed the cellular components within CS. Using fluorescence-activated cell sorting (FACS) of dissociated CS, we found that CPCs (Sca-1^+^/CD45^−^ subpopulation) made up a large fraction of CS cells (~25%) in young mice, both at baseline and post-MI (Figure 2). However, the proportion of Sca-1^+^/CD45^−^ CPCs in CSs from aging mice was significantly lower than those from young at baseline and 1-week post-MI (*p* < 0.001) (Figure 2). Cells expressing c-kit^+^, CD133^+^, CD34^+^, Flk1^+^ and CD31^+^ in CSs were rare (less than 2%) and were not significantly different in CSs from young and aging mice.

### 3.2. Impaired Cardiac Fibroblast Proliferation with Age

**Figure 3:**
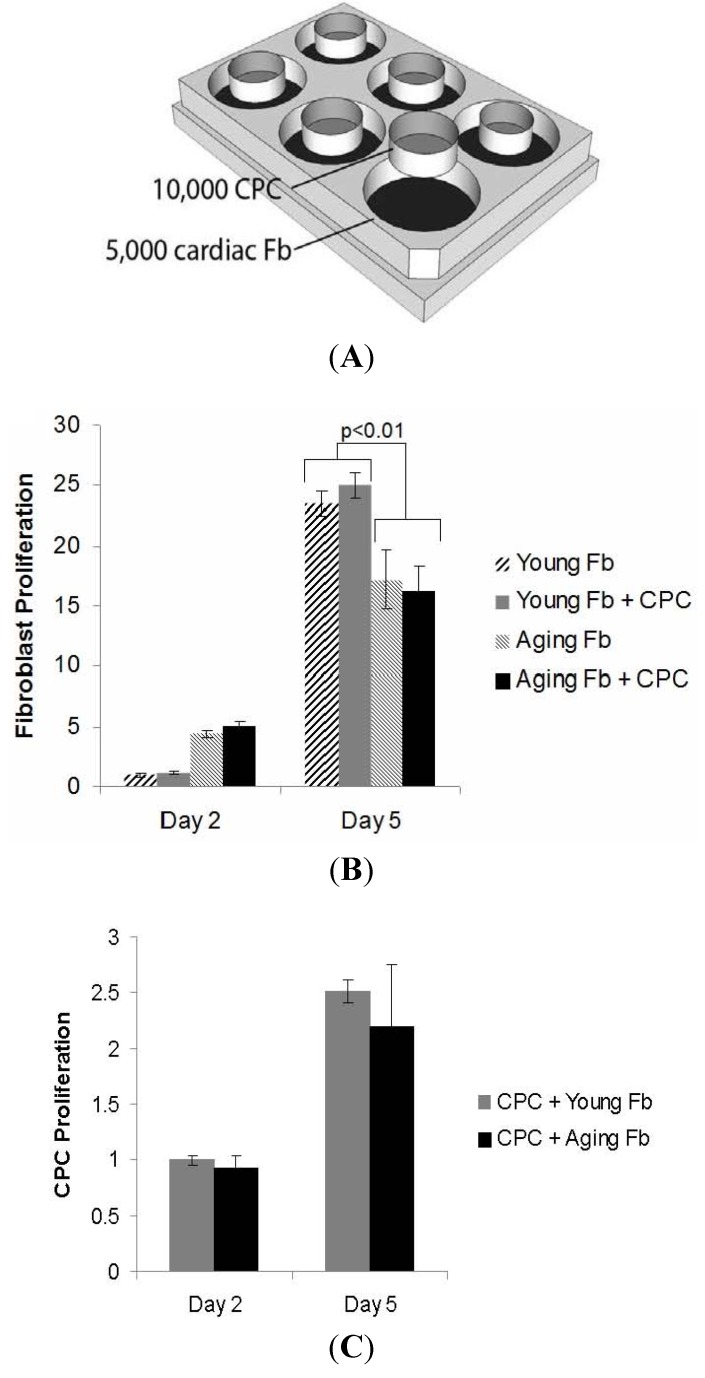
Impaired proliferation of aging cardiac fibroblasts is independentof the presence of CPCs.In this experiment we plated fibroblasts in the well and the CPCs in the inserts. (**A**) There were more aging fibroblasts early, but the growth rate was slower thanthe young fibroblasts. This was unaffected by the presence of CPCs ((**B**), *n* = 4 pergroup). The CPCs in the insert proliferated to the same extent in the presence ofyoung and aging fibroblasts ((**C**), *n* = 4 per group).

To assess the reason for slower outgrowth from cardiac explants with age, we cultured aging cardiac fibroblasts and compared their growth rates compared to young cardiac fibroblasts. Aging cardiac fibroblasts proliferated more slowly than the young fibroblasts (*p* < 0.01; Figure 3). To simulate the growth of cardiospheres, where CPCs grow alongside cardiac fibroblasts, we co-cultured cardiac fibroblasts with CPCs. The rate of proliferation of cardiac fibroblasts was unaffected by the presence or absence of CPCs (Figure 3), indicating no paracrine signaling effect on fibroblast proliferation.

### 3.3. Aging Fibroblasts Have No Paracrine Signaling Effect on CPC Proliferation

**Figure 4:**
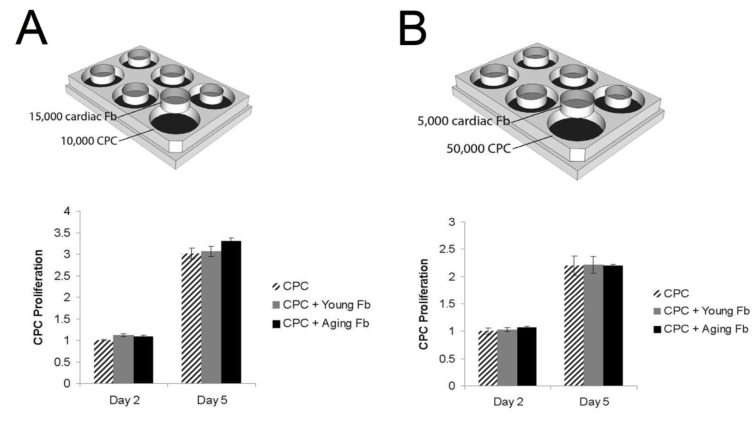
Proliferation of Sca-1^+^/CD45^−^ cardiac progenitor cells at low (**A**) and high (**B**) seeding density. 1 × 10^4^ CPCs were seeded into each well and 1.5 × 10^4^ cardiac fibroblasts were seeded into each insert (**A**). There was no difference in CPC proliferation when cultured with young or aging fibroblasts compared to cell free medium (*n* = 4 per group). To exclude an effect of cell density, we repeated the experiment with 5 × 10^4^ CPCs seeded into each well and 5 × 10^3^ cardiac fibroblasts seeded into each insert (**B**). CPCs proliferated at the same rate regardless of the presence of young or aging fibroblasts (*n* = 4 per group). Fb = fibroblasts; CPC = cardiac progenitor cells.

CPCs were plated in non-contact co-culture with cardiac fibroblasts to determine the effect of the fibroblasts on CPC proliferation. The number of viable CPCs approximately doubled from day 3 to day 5 in culture. At both time points, no differences in proliferation rate were detected when co-cultured with young or aging cardiac fibroblasts compared to cell-free medium (Figure 4). To confirm our data and exclude an effect of cell dose, we repeated the experiment using different cell density. In the first experiment, cardiac fibroblasts were in excess of CPCs, and in the second experiment the CPCs were in higher numbers than cardiac fibroblasts (Figure 4). In both cases, we found no paracrine effect from the co-cultured fibroblasts on the proliferation rate of CPCs.

## 4. Discussion

Our study has several important findings. First, we show an age-related decrease in the growth rate of CS, and a blunting of the proliferative capacity after MI. Second, we found that CPCs made up a much smaller proportion of CS with age. Third, we show that aging is associated with less proliferative capacity in cardiac fibroblasts. Fourth, we demonstrated a lack or paracrine interaction between cardiac fibroblasts and CPCs in the proliferation of either cell type. These results have important implications for clinical trials of autologous cell therapy in aging patients following MI.

Cell therapy shows promise for ameliorating heart failure following MI. MI and heart failure become more prevalent with increasing age [14]. Disappointingly, however, the results of cell therapy in experimental MI are less robust in the aged compared to the results achieved in the young. The inferior results with aging have been attributed to the age of the cells delivered [15,16,17,18], but may also relate to the age of the recipient tissue [19]. A more in depth understanding of the mechanism by which cell therapy is less effective in aging remains elusive. One aspect of autologous CS or CPC therapy that may limit its uptake clinically is the ability to generate enough cells for therapy in the aging population. Our data demonstrate that aging significantly reduces the number of CPCs available for cell therapy. This does not appear to be a problem of just reduced CS growth from slower explant outgrowth, or impairment of cardiac fibroblast proliferation. The low percentage of CPCs in CS demonstrates that there is a defect in proliferation of the CPCs themselves. Much of the early experience with human CPCs has been from biopsies taken from transplanted hearts, which tend to be younger and healthier than many post-MI patients. It is enticing to speculate that more biopsy tissue and longer culture times may overcome this issue in aging patients, but this should be tested in future clinical studies in aging patients. It is noteworthy that we used 18-month-old mice as our aging group. We chose this age because we have previously demonstrated a detectable increase in fibrosis and reduction in cardiac function at this age [20]. These are not very elderly senescent mice, but more middle-aged, as C57/Bl6 live to approximately 30 months of age in captivity [21]. One could reasonably expect further reductions in proliferative capacity as the mice age further. Our data support the use of allogeneic CPCs from young donors for cell therapy aimed at myocardial regeneration. 

Aging has significant effects on post-infarction LV remodeling. We have extensively reviewed this topic [3]. Several differences of the aging heart are germane to the findings of this study. First, the aging heart has increased fibrosis at baseline [20], yet the cardiac fibroblasts derived from aging hearts have impaired proliferative capacity. Cieslik and colleagues studied fibroblasts from aging hearts [22] and found that, compared to those derived from young hearts, they had impaired response to transforming growth factor beta (TGFb) but had increased collagen production both at baseline and in response to insulin. Their demonstration of reduced responsiveness of aging cardiac fibroblasts to proliferative factors is congruent with our data, and is also in keeping with the literature regarding fibrosis in the aging heart [20]. Furthermore, TGFb is necessary for formation of CS [23]. One could speculate that reduced TGFb signaling could be a common cause for both the dysfunctional CS and the hypoproliferative fibroblasts in the aging heart, but this remains to be tested in future studies.

Our results have implications beyond CS. The interactions, or lack thereof, have wider consequences for our understanding of CPCs residing in the stem cell niche *in vivo*. The cardiac stem cell niche consists of CPCs, cardiomyocytes, fibroblasts, and other supporting cells [24,25]. It is an intricate three-dimensional structure that provides a specific microenvironment for the maintenance of CPCs. When injury occurs, CPCs can be triggered to mobilize and differentiate into other cell types as needed. We show that there is no effect of the presence of fibroblasts on the proliferation of CPCs. This demonstrates that the defect in CPC proliferation with age is a cell-autonomous problem, rather than an environmental effect from the aging cells surrounding the CPCs. The ratio of CPCs to fibroblasts may influence the amount of secreted molecules, which in turn may affect the level of response. The adult murine heart is made up of about 25% fibroblasts, but CPCs are scarce [9]. Furthermore, the ratio is likely to be different within the stem cell niche. To address the issue of relative cell concentration, we performed our experiments with CPCs in excess and again with fibroblasts in excess, and found cell numbers had no effect. The repeated trials using different cell ratios and growth surfaces demonstrate that the observed result was not merely a dose-response or attachment problem. We recognize two potential limitations to this study; first, our *in vitro* experiments may create a niche that is different from the *in vivo* structure and composition of the cardiac stem cell niche. However, we believe it is important to start with single cell interactions before attempting to recapitulate the entire niche *in vitro.* Second, there may be important interactions between CPCs and fibroblasts that require direct cell-cell contact, such as signaling through gap junctions. This topic requires further study in direct cell-cell contact culture conditions.

## 5. Conclusions

In conclusion, aging hearts generate fewer CPCs, and aging CPCs have significantly reduced proliferative potential following MI. Aging cardiac fibroblasts also have reduced proliferative capacity, but these appear to be cell-autonomous problems, not caused by paracrine signaling between cell types, as cardiac fibroblasts do not affect the proliferation of CPCs, and CPCs cannot rescue the impaired proliferation of aging fibroblasts.

## References

[1] National Heart Lung and Blood Institute (2006). Incidence and Prevalence. 2006 Chart Book on Cardiovascular and Lung Diseases.

[2] Maggioni A.A., Maseri A., Fresco C., Franzosi M.G., Mauri F., Santoro E., Tognoni G. (1993). The investigators of the gruppo italiano por lo studio della sopravvivenza nell’infarto M: Age-related increase in mortality among patients with first myocardial infarctions treated with thrombolysis. N. Engl. J. Med..

[3] Shih H., Lee B., Lee R.J., Boyle A.J. (2011). The aging heart and post-infarction left ventricular remodeling. J. Am. Coll. Cardiol..

[4] Anversa P., Rota M., Urbanek K., Hosoda T., Sonnenblick E., Leri A., Kajstura J., Bolli R. (2005). Myocardial aging—A stem cell problem. Bas. Res. Cardiol..

[5] Ye J., Boyle A.J., Shih H., Sievers R.E., Zhang Y., Prasad M., Su H., Zhou Y., Grossman W., Bernstein H.S. (2012). Sca-1^+^ cardiosphere-derived cells are enriched for isl1-expressing cardiac precursors and improve cardiac function after myocardial injury. PLoS One.

[6] Ye J., Boyle A., Shih H., Sievers R., Wang Z.-E., Gormley M., Yeghiazarians Y. (2013). CD45 positive cells are not an essential component in cardiosphere formation. Cell Tissue Res..

[7] Li T.-S., Cheng K., Lee S.-T., Matsushita S., Davis D., Malliaras K., Zhang Y., Matsushita N., Smith R.R., Marbán E. (2010). Cardiospheres recapitulate a niche-like microenvironment rich in stemness and cell-matrix interactions, rationalizing their enhanced functional potency for myocardial repair. Stem Cells.

[8] Makkar R.R., Smith R.R., Cheng K., Malliaras K., Thomson L.E.J., Berman D., Czer L.S.C., Marban L., Mendizabal A., Johnston P.V. (2012). Intracoronary cardiosphere-derived cells for heart regeneration after myocardial infarction (CADUCEUS): A prospective, randomised phase 1 trial. Lancet.

[9] Banerjee I., Fuseler J.W., Price R.L., Borg T.K., Baudino T.A. (2007). Determination of cell types and numbers during cardiac development in the neonatal and adult rat and mouse. Am. J. Physiol. Heart Circ. Physiol..

[10] Smith R.R., Barile L., Cho H.C., Leppo M.K., Hare J.M., Messina E., Giacomello A., Abraham M.R., Marban E. (2007). Regenerative potential of cardiosphere-derived cells expanded from percutaneous endomyocardial biopsy specimens. Circulation.

[11] Lindsey M.L., Goshorn D.K., Squires C.E., Escobar G.P., Hendrick J.W., Mingoia J.T., Sweterlitsch S.E., Spinale F.G. (2005). Age-dependent changes in myocardial matrix metalloproteinase/tissue inhibitor of metalloproteinase profiles and fibroblast function. Cardiovasc. Res..

[12] Messina E., de Angelis L., Frati G., Morrone S., Chimenti S., Fiordaliso F., Salio M., Battaglia M., Latronico M.V.G., Coletta M. (2004). Isolation and expansion of adult cardiac stem cells from human and murine heart. Circ. Res..

[13] Yeghiazarians Y., Zhang Y., Prasad M., Shih H., Saini S.A., Takagawa J., Sievers R.E., Wong M.L., Kapasi N.K., Mirsky R. (2009). Injection of bone marrow cell extract into infarcted hearts results in functional improvement comparable to intact cell therapy. Mol. Ther..

[14] Roger V.L., Go A.S., Lloyd-Jones D.M., Benjamin E.J., Berry J.D., Borden W.B., Bravata D.M., Dai S., Ford E.S., Fox C.S. (2012). Heart disease and stroke statistics—2012 update. A report from the American Heart Association. Circulation.

[15] Wang X., Takagawa J., Haddad D.J., Pinnamaneni K., Zhang Y., Sievers R.E., Grossman W., Yeghiazarians Y., Springer M.L. (2011). Advanced donor age impairs bone marrow cell therapeutic efficacy for cardiac disease. J. Tissue Sci. Eng..

[16] Fan M., Chen W., Liu W., Du G.-Q., Jiang S.-L., Tian W.-C., Sun L., Li R.-K., Tian H. (2010). The effect of age on the efficacy of human mesenchymal stem cell transplantation after a myocardial infarction. Rejuv. Res..

[17] Khan M., Mohsin S., Khan S.N., Riazuddin S. (2011). Repair of senescent myocardium by mesenchymal stem cells is dependent on the age of donor mice. J. Cell. Mol. Med..

[18] Pallante B.A., Duignan I., Okin D., Chin A., Bressan M.C., Mikawa T., Edelberg J.M. (2007). Bone marrow Oct3/4^+^ cells differentiate into cardiac myocytes via age-dependent paracrine mechanisms. Circ. Res..

[19] Kan C.-D., Li S.-H., Weisel R.D., Zhang S., Li R.-K. (2007). Recipient age determines the cardiac functional improvement achieved by skeletal myoblast transplantation. J. Am. Coll. Cardiol..

[20] Boyle A.J., Shih H., Hwang J., Ye J., Lee B., Zhang Y., Kwon D., Jun K., Zheng D., Sievers R. (2011). Cardiomyopathy of aging in the mammalian heart is characterized by myocardial hypertrophy, fibrosis and a predisposition towards cardiomyocyte apoptosis and autophagy. Exp. Gerontol..

[21] Yuan R., Tsaih S.-W., Petkova S.B., Evsikova C.M.D., Xing S., Marion M.A., Bogue M.A., Mills K.D., Peters L.L., Bult C.J. (2009). Aging in inbred strains of mice: Study design and interim report on median lifespans and circulating IGF1 levels. Aging Cell.

[22] Cieslik K.A., Trial J., Carlson S., Taffet G.E., Entman M.L. (2013). Aberrant differentiation of fibroblast progenitors contributes to fibrosis in the aged murine heart: Role of elevated circulating insulin levels. FASEB J..

[23] Forte E., Miraldi F., Chimenti I., Angelini F., Zeuner A., Giacomello A., Mercola M., Messina E. (2012). TGFβ-dependent epithelial-to-mesenchymal transition is required to generate cardiospheres from human adult heart biopsies. Stem Cells Dev..

[24] Leri A., Kajstura J., Anversa P. (2005). Cardiac stem cells and mechanisms of myocardial regeneration. Physiol. Rev..

[25] Urbanek K., Cesselli D., Rota M., Nascimbene A., de Angelis A., Hosoda T., Bearzi C., Boni A., Bolli R., Kajstura J. (2006). Stem cell niches in the adult mouse heart. PNAS.

